# *Helicobacterpylori* eradication following endoscopic resection might prevent metachronous gastric cancer: a systematic review and meta-analysis of studies from Japan and Korea

**DOI:** 10.3389/fmed.2024.1393498

**Published:** 2024-09-02

**Authors:** Tian-Hang Yu, Dan Bai, Kai Liu, Wei-Han Zhang, Xin-Zu Chen, Jian-Kun Hu

**Affiliations:** ^1^Gastric Cancer Center & Gastric Cancer Laboratory, Department of General Surgery, West China Hospital, Sichuan University, Chengdu, China; ^2^Department of Day Surgery, General Practice Medical Center, West China Hospital, Sichuan University, Chengdu, China; ^3^Ya’an Cancer Prevention and Control Center, Ya’an People’s Hospital – West China Ya’an Hospital, Sichuan University, Ya’an, China; ^4^Ya’an Key Laboratory for High Altitude Medicine, Ya’an People’s Hospital - West China Ya’an Hospital, Sichuan University, Ya’an, China

**Keywords:** early gastric cancer, metachronous gastric cancer, *Helicobacter pylori*, endoscopic resection, eradication

## Abstract

**Objectives:**

A systematic review and meta-analysis was performed to evaluate the preventive effectiveness of *Helicobacter pylori* eradication against metachronous gastric cancer (MGC) or dysplasia following endoscopic resection (ER) for early gastric cancer (EGC) or dysplasia.

**Methods:**

PubMed, Cochrane Library, MEDLINE, and EMBASE were searched until 31 October 2023, and randomized controlled trials or cohort studies were peer-reviewed. The incidence of metachronous gastric lesions (MGLs) including MGC or dysplasia was compared between *Helicobacter pylori* persistent and negative groups, eradicated and negative groups, and eradicated and persistent groups.

**Results:**

Totally, 21 eligible studies including 82,256 observations were analyzed. Compared to those never infected, *Helicobacter pylori* persistent group (RR = 1.58, 95% CI = 0.98–2.53) trended to have a higher risk of MGLs and significantly in partial subgroups, while the post-ER eradicated group (RR = 0.79, 95% CI = 0.43–1.45) did not increase the risk of MGLs. Moreover, successful post-ER eradication could significantly decrease the risk of MGLs (RR = 0.54, 95% CI = 0.44–0.65) compared to those persistently infected. Sensitivity analysis obtained generally consistent results, and no significant publication bias was found.

**Conclusion:**

The persistent *Helicobacter pylori* infection trends to increase the post-ER incidence of MGC or dysplasia, but post-ER eradication can decrease the risk correspondingly. Post-ER screening and eradication of *Helicobacter pylori* have preventive effectiveness on MGC, and the protocol should be recommended to all the post-ER patients.

**Systematic review registration**: The PROSPERO registration identification was CRD42024512101.

## Introduction

Gastric cancer is the fifth most common cancer and the fourth most common cause of cancer death globally, according to 768,793 deaths in 2020 (1, 2). Epidemiologic and clinical studies indicate that the hotspot of incidence and mortality events of gastric cancer exists in East Asia, probably due to the differences in population-specific genetic risk factors and infectious agents such as *Helicobacter pylori* (*H. pylori*) (3–7).

Gastrectomy with lymphadenectomy was regarded as the standard treatment for gastric cancer (8–10). In recent decades, endoscopic resection (ER) including endoscopic mucosal resection (EMR) and endoscopic submucosal dissection (ESD) for early gastric cancer (EGC) has been widely accepted as curative therapy (8). Current clinical evidence suggests that long-term survival after ER for EGC is comparable to surgical resection (11–13). Additionally, patients who underwent ER might have a higher risk of metachronous gastric cancer (MGC) compared with those who underwent gastrectomy, but MGC after ER was successfully re-treated without affecting overall survival (14).

In 1975, Correa et al. first reported the astute observation that intestinal-type gastric adenocarcinoma was associated with an inflammatory process in the stomach, namely Correa’s cascade: normal gastric mucosa, non-atrophic gastritis, atrophic gastritis, intestinal metaplasia, intraepithelial neoplasia (dysplasia), and then gastric cancer (15). Later reports showed that *H. pylori*, as a particular bacterial species, could colonize the stomach and initiate an inflammatory response or atrophic gastritis, and therefore *H. pylori* infection was the most well-described risk factor for non-cardia gastric cancer (16–19).

However, the exact role of *H. pylori* infection in the development of post-ER metachronous gastric lesions (MGL) including late-stage precancerous lesion (dysplasia) or MGC has not been clearly elucidated. Some studies indicated that post-ER *H. pylori* eradication could reduce the risk of MGC, but a few suggested it was not worthy instead (20, 21). Thus, we aimed to conduct a systematic review and meta-analysis to evaluate the association between *H. pylori* status and the risk of MGLs. We hypothesized that (A) post-ER persistent infection of *H. pylori* might increase the risk of MGLs and then (B) successful post-ER eradication of *H. pylori* might decrease the risk of MGLs.

## Methods

### Literature search

PubMed, Cochrane Library, MEDLINE, and EMBASE were searched until 31 October 2023. The search strategy combined the following MESH items: *Helicobacter pylori*; Endoscopy; Gastrointestinal, Neoplasms, Second Primary. The synonyms of these items were also included in the search strategy, such as *Helicobacter nemestrinae*, *Campylobacter pylori*, Endoscopic Gastrointestinal Surgery, Neoplasms, Metachronous, Second Malignancy. The search link in PubMed was shown in Supplementary materials. We mainly used PubMed, and the same search strategy was used in the Cochrane Library, MEDLINE, and EMBASE databases as supplements.

### Eligibility

Either randomized controlled trials (RCTs) or cohort studies were potentially eligible. ERs were performed for EGC or dysplasia. The outcome of post-ER *H. pylori* eradication on the prevention of MGLs was compared to negative controls. The status of *H. pylori* infection was examined in all patients by any possible test, and they were classified into three categories: (A) *H. pylori*–negative group, (B) *H. pylori*-eradicated group, and (C) *H. pylori*-persistent group. The *H. pylori*-negative group consisted of patients who was never detected before the ER and were negative during follow-up. The *H. pylori*-eradicated group consisted of patients who were diagnosed with *H. pylori* infection at or before the time of ER and received eradication therapy, with no evidence of *H. pylori* infection after the re-examination during follow-up. The *H. pylori*-persistent group consisted of patients who remained *H. pylori* positive during follow-up regardless of eradication or not. Endoscopic follow-up was performed at the post-ER 1-year visit or later. The outcome measure was defined as the incidence of MGLs, including the subsets (A) MGC and dysplasia or (B) dysplasia only. There was no limitation on publication date, language, or country. If the outcome data were unextractable, the studies were excluded.

### Selection, assessment, and data extraction

The eligibility of literature was peer-reviewed, and any disagreement was resolved through discussion between peer-reviewers or arbitration by a third party. Quality assessment was carried out using the Cochrane risk-of-bias tool for RCT and Newcastle–Ottawa scale for observational studies (22, 23). The general information of eligible studies was extracted including the first author, publication year, country, primary disease, endoscopic intervention, *H. pylori* test, eradication regimen, and follow-up duration. Besides, the number of observed participants and the number of MGL events were extracted or estimated in each group.

### Statistics

This meta-analysis was conducted using the R Studio software with the R package “meta.” The risk ratio (RR) and 95% confidence interval (CI) were estimated as the effect size. Between-study heterogeneity was assessed by I-square and Cochran’s Q. A fixed-effects model was used for those with I-square value of <50%, or a random-effects model was used instead. The forest plots were presented to display the meta-analysis. Subgroup analysis was conducted with regard to the study design and primary outcome. Publication bias was first estimated using the funnel plot and then confirmed using the Egger’s test, the AS-Thompson test, the Duval and Tweedie trim-and-fill method, the contour-enhanced meta-analysis funnel plot, and the Baujat plot, where applicable (24–26). A *p*-value of <0.05 was considered statistically significant.

Comparisons were conducted between persistent and negative groups for hypothesis A, as well as between eradicated and negative groups as secondary analyses. Comparisons were conducted between persistent and eradicated groups for hypothesis B. Subgroup analyses were carried out regarding the subsets of study designs, countries, primary diseases, and outcome measures. Sensitive analysis by the leave-one-out method was also performed to evaluate whether any study had excessive influence on the results of the pooled analysis.

### Reporting

This systematic review and meta-analysis was conducted according to the Meta-analysis Of Observational Studies in Epidemiology (MOOSE) 2000 statements (27), and a flow diagram was drawn in accordance with the Preferred Reporting Items for Systematic Reviews and Meta-Analysis (PRISMA) guidelines (28).

### Registration

The present meta-analysis was registered in the PROSPERO International Prospective Register of Systematic Reviews supported by the National Institute for Health Research of the National Health Service (NHS), UK (ID: CRD42024512101) (29).

### Ethics

Ethical approval was not required due to the nature of literature-based research.

## Results

### Study information

**Figure 1:**
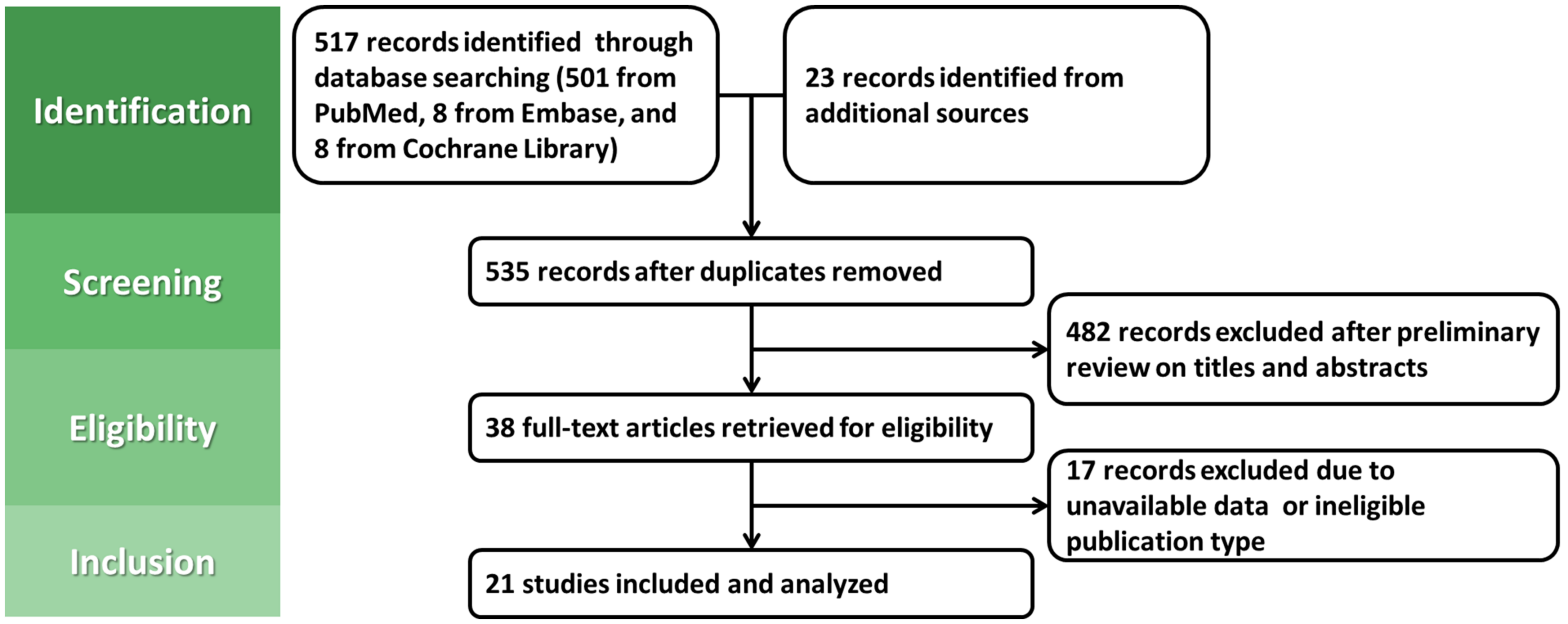
The PRISMA flow diagram of the meta-analysis.

The flow diagram of study selection is presented in Figure 1. Finally, 21 studies were included for the meta-analysis, including 3 RCTs (30–32) and 18 cohort studies (20, 21, 33–48). The brief characteristics of the included studies are summarized in Supplementary Table S1. Due to the lack of relevant research in European and American countries, all the included studies were from Japan and Korea. All the included studies were of high or moderate quality (Supplementary Table S2, S3). Among the 21 studies, MGLs developed in 1,233 out of 38,931 eradicated patients and 2,982 out of 41,969 persistent patients, compared with 92 out of 1,356 negative patients. Additionally, among those with dysplasia only receiving post-ER eradication therapy, 917 out of 34,494 eradicated patients and 2,469 out of 36,059 persistent patients found MGLs.

### *Helicobacter pylori* infection and MGL risk

**Figure 2:**
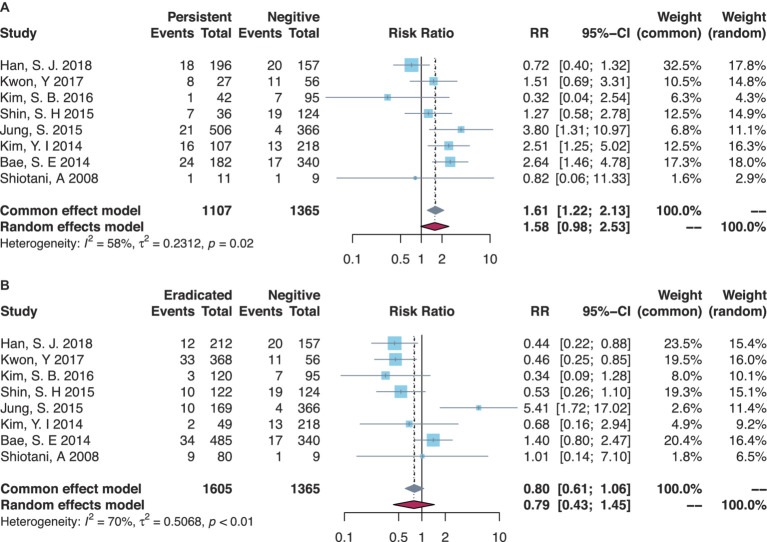
Forest plots of overall comparisons **(A)** between *H. pylori*-persistent and negative patients and **(B)** between eradicated and negative patients.

There were eight studies that compared the MGL risk between *H. pylori*-persistent and negative statuses, while the risk trended to be increased in the persistent group but not significantly yet (RR = 1.58, 95% CI = 0.98–2.53, I-square = 58%) (Table 1, Figure 2A). Subgroup analyses are shown in Table 1 and Supplementary Figures S1–S8. The majority of Korean studies obtained consistent results (RR = 1.61, 95% CI = 0.99–2.61, I-square = 63%). In the primary disease subsets of EGC combining dysplasia or not, higher risks of MGLs were found in the persistent group than those in the negative group (RR = 3.80, 95% CI = 1.31–10.97). Similarly, in the MGL outcome subsets of MGC combining dysplasia or not, the persistent group had a higher risk of MGL events (RR = 1.93, 95% CI = 1.17–3.17). Besides, the secondary analyses by comparing the eradicated group to the negative group demonstrated generally comparable risks of post-ER MGLs (Table 1, Figure 2B).

**Table 1:** Meta-analyses on associations between *H. pylori* infection and MGL risk.

Subsets	Persistent vs. negative			Eradicated vs. negative		
	Study count	RR (95% CI)	*p*-value	Study count	RR (95% CI)	*p*-value
Total	8	1.58 (0.98–2.53)^a^	0.06	8	0.79 (0.43–1.45)^a^	0.44
Study design						
RCT only	0	N/A	N/A	0	N/A	N/A
Cohort only	8	1.58 (0.98–2.53)^a^	0.06	8	0.79 (0.43–1.45)^a^	0.21
Country						
Japan	1	0.82 (0.06–11.33)	0.88	1	1.01 (0.14–7.10)	0.99
Korea	7	1.61 (0.99–2.61)^a^	0.06	7	0.78 (0.40–1.50)^a^	0.45
Primary disease						
EGC or dysplasia	1	3.80 (1.31–10.97)	0.01	1	5.41 (1.72–17.01)	<0.01
EGC only	6	1.42 (0.79–2.57)^a^	0.24	6	0.64 (0.37–1.10)^a^	0.10
Dysplasia only	1	1.27 (0.58–2.78)	0.55	1	0.53 (0.26–1.10)	0.09
MGL outcome						
MGC or dysplasia	3	1.93 (1.17–3.17)	0.01	3	1.03 (0.23–4.61)^a^	0.97
MGC only	5	1.35 (0.63–2.92)^a^	0.44	5	0.71 (0.37–1.35)^a^	0.29
Dysplasia only	0	N/A	N/A	0	N/A	N/A

CI, confidence interval; EGC, early gastric cancer; MGC, metachronous gastric cancer; MGL, metachronous gastric lesion; N/A, not applicable; RCT, randomized controlled trial; RR, risk ratio.

a Random-effects model was used due to heterogeneity.

### Preventive effectiveness of post-ER eradication

**Figure 3:**
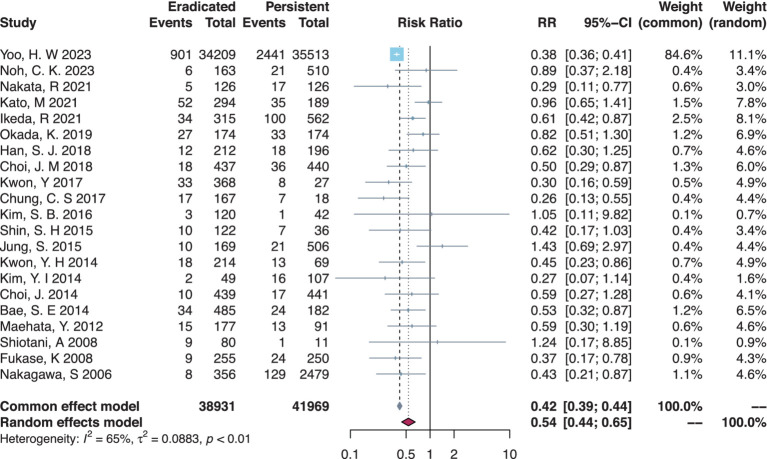
Forest plot of the overall comparison between *H. pylori*-eradicated and persistent patients.

There were 21 studies that compared the MGL risk between *H. pylori*-eradicated and persistent statuses, and post-ER eradication had a significant protective effect by lowering the MGL risk (RR = 0.54, 95% CI = 0.44–0.65, I-square = 65%) (Table 2, Figure 3). In subgroup analysis (Table 2, Supplementary Figures S9–S12), meta-analyses on RCTs or cohort studies did not differ in results. Either Japanese or Korean studies obtained consistent results, respectively. In the primary disease subset of either EGC only or dysplasia only, similar protective effects against MGLs were found in the eradicated group compared to the persistent group. Additionally, in the MGL outcome subsets of MGC combining dysplasia or not, the eradicated group had a significantly lower risk of MGLs.

**Table 2:** Meta-analyses on the preventive effectiveness of post-ER *H. pylori* eradication.

Subsets	Eradicated vs. Persistent		
	Study count	RR (95% CI)	*p*-value
Total	21	0.54 (0.44–0.65)^a^	<0.01
Study design			
RCT only	3	0.48 (0.33–0.70)	<0.01
Cohort only	18	0.55 (0.44–0.68)^a^	<0.01
Country			
Japan	8	0.63 (0.52–0.77)	<0.01
Korea	13	0.40 (0.37–0.42)	<0.01
Primary disease			
EGC or dysplasia	3	0.73 (0.39–1.38)^a^	0.33
EGC only	15	0.57 (0.49–0.68)	<0.01
Dysplasia only	3	0.39 (0.36–0.42)	<0.01
MGL outcome			
MGC or dysplasia	8	0.49 (0.33–0.70)^a^	<0.01
MGC only	13	0.60 (0.51–0.71)	<0.01
Dysplasia only	0	N/A	N/A

CI, confidence interval; EGC, early gastric cancer; MGC, metachronous gastric cancer; MGL, metachronous gastric lesion; N/A, not applicable; RCT, randomized controlled trial; RR, risk ratio.

a Random-effects model was used due to heterogeneity.

### Sensitivity analysis

Through leave-one-out analyses, consistent results were found in all the three principal meta-analyses, including the comparisons between *H. pylori*-persistent and negative groups, eradicated and negative groups, and eradicated and persistent groups (data not shown).

### Publication bias

**Figure 4:**
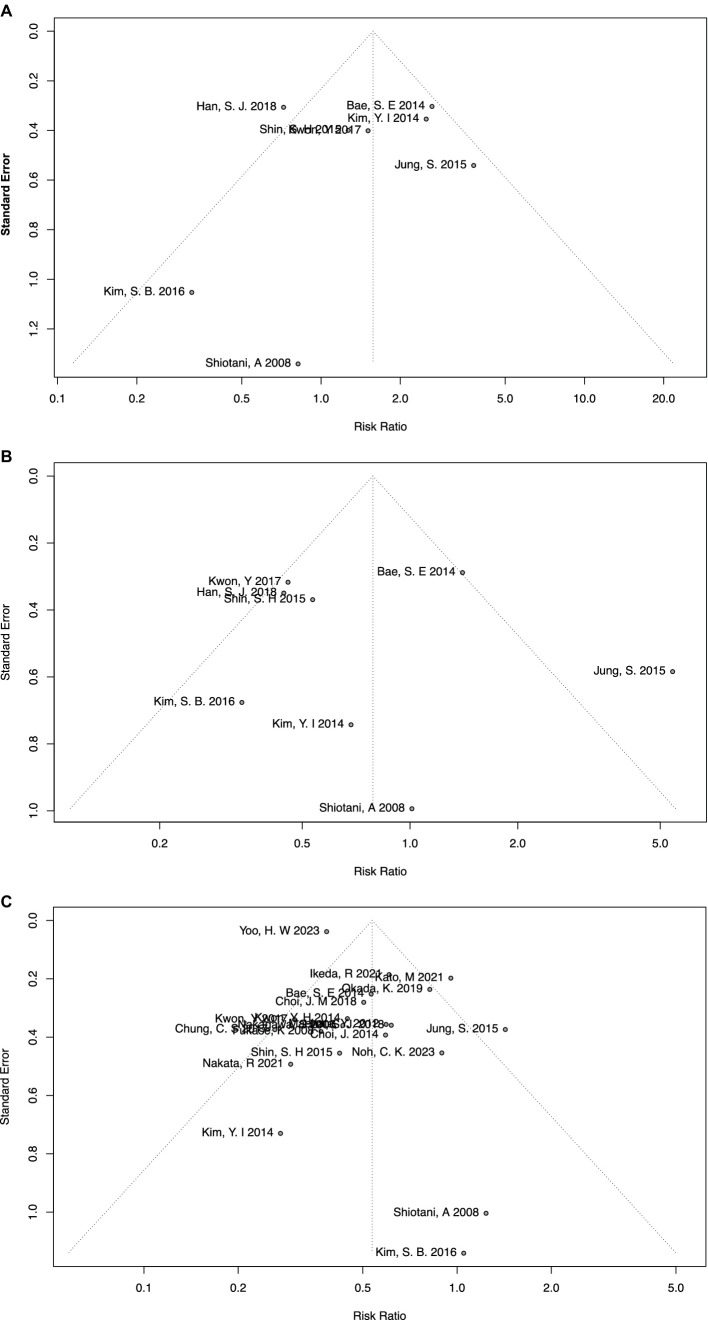
Funnel plots of meta-analyses on overall comparisons **(A)** between persistent and negative patients, **(B)** between eradicated and negative patients, and **(C)** between eradicated and persistent patients.

In the meta-analysis of *H. pylori*-persistent and negative groups, no significant publication bias was found using the funnel plot (Figure 4A) or Egger’s test (*p* = 0.63). Similarly, no significant publication bias was found in the meta-analysis of eradicated and negative groups using the funnel plot (Figure 4B) or Egger’s test (*p* = 0.78).

In particular, the meta-analysis of eradicated and persistent groups included more than 10 studies, and therefore the AS-Thompson test was used to test the potential sources of high heterogeneity. No significant publication bias was tested (*p* = 0.24), along with the funnel plot (Figure 4C). Additionally, the Duval and Tweedie trim-and-fill method and contour-enhanced meta-analysis funnel plots were also applied to visually depict publication bias. By using the Duval and Tweedie trim-and-fill method, seven more studies were included and all the added studies were located in the *p* < 0.05 area of the contour-enhanced meta-analysis funnel plots, namely, null publication bias (Supplementary Figure S13). Moreover, the Duval and Tweedie trim-and-fill method was also used as part of sensitivity analysis, which suggested consistent and reliable results (RR = 0.41, 95% CI = 0.32–0.53, I-square = 75%). Finally, the Baujat plot indicated that the study by Yoo et al. had the greatest impact on the overall result (33), and the study by Kato et al. had the greatest impact on heterogeneity (35).

## Discussion

This system review and meta-analysis analyzed 21 studies with 82,256 observations. *H. pylori*-persistent group trended to have a higher risk of MGLs compared to those never infected, but not in the post-ER eradicated group. Moreover, successful post-ER eradication could decrease the risk of MGLs compared to those persistently infected. Sensitivity analysis obtained generally consistent results, and no significant publication bias was found. However, heterogeneity commonly existed among meta-analyses, and a random-effects model was used accordingly. In the bibliographic view, the present report might be a most updated meta-analysis.

Gastric cancer is still one of the top malignancies in China, with a heavy burden of high population incidence (19, 49). *H. pylori* infection is one of the most important risk factors for gastric cancer in East Asia, particularly in China (50, 51). A multicenter prospective cohort study including 512,715 Chinese indicates that *H. pylori* infection accounted for 78.5% of non-cardia and 62.1% of cardia cancers, and up to 339,955 incident gastric cancers could be attributable to *H. pylori* infection (52). Due to consistent advocacy, health education, improved sanitary condition, and drinking water quality in China, the *H. pylori* infection rate among Chinese has slowly declined over the past 30 years, especially among the urban health check-up population (50, 53). Active *H. pylori* eradication within organized massive screening might simultaneously lower the incidence and improve population survival of gastric cancer (54).

Currently, a low proportion of EGC, usually no more than 20%, is the principal difficulty in improving the population survival of gastric cancer in China (55). In contrast, the proportions of EGC are fairly high in both Japan and Korea due to national organized massive screening for gastric cancer. Therefore, ER as a minimally invasive procedure is well-practiced for the early treatment of EGC, with a negligible risk of lymph node metastasis among high-selected candidates. According to Japanese Gastric Cancer Treatment Guidelines 2021 (6th edition), the EMR or ESD absolute indications for EGC include a differentiated-type adenocarcinoma without ulcerative findings in which the depth of invasion is clinically diagnosed as T1a and the maximal size is ≤ 2 cm (56). The experiences of post-ER management in Japan and Korea should be much substantial, and thus the present meta-analysis pooled studies just available from Japan and Korea.

At the 1-year or later endoscopic follow-up after ER or gastrectomy for EGC, the MGC has been more prone to occur after ER instead of gastrectomy (57). It may be due to the fact that the stomach can be preserved after ER, while most of the stomach is removed postgastrectomy with a limited area of mucosa left. Meanwhile, some studies have shown that conventional mucosal resection has a higher incidence of post-ER MGC than expanded mucosal resection (58). However, providing repeated ER treatment, there is no significant difference in long-term survival outcomes between the expanded and conventional groups (58). Besides, the other potential risk factors for MGC after ER for EGC included male, older age, severe or corpus intestinal metaplasia, family history of gastric cancer, synchronous adenoma, and *H. pylori* infection (59–61). It is the rationale why the present meta-analysis hypothesizes *H. pylori* infection could increase MGC risk and post-ER eradication might benefit in preventing MGC.

Regarding *H. pylori* infection, some consider eradication is no longer meaningful since gastric cancer has already developed, but investigation has shown that after eradication, the richness and uniformity of the gastric microbiota could be restored to a state similar to that of negative subjects. From bench to bedside, our updated findings evidence the capacity of reducing MGL incidence by post-ER eradication with plausible reporting quality, consistent with previous studies (62–64). Some studies suggested that the effectiveness of eradication could be observed by decreasing MGC incidence after the 6th post-ER year, but not at 3-year follow-up (31). Specifically, adequate length of endoscopic surveillance must be quite important in common practice, and diverse follow-up situations might introduce the source of heterogeneity in the present meta-analysis. Additionally, the time interval between the first ER and *H. pylori* eradication needs to be taken into account. As reported in the study by Kato et al. (35), all the patients were required to undergo *H. pylori* eradication within no more than 1 year after ER. Moreover, the regimens of *H. pylori* eradication are unspecific to post-ER patients. Actually, the risk factors of MGLs and the best follow-up strategy are still underestimated up to the known knowledge.

### Limitation

Some limitations need attention yet. First, all the included studies came from Japan and Korea, and the extrapolation of results kept unclear to other high-risk areas. Second, although the quality of the included cohort studies was high or moderate, only three available RCTs indicated that selection and performance biases were inevitable. Third, in many studies, follow-up work was not long enough and without regular endoscopic surveillance, which could introduce between-study heterogeneity. Fourth, the time interval between *H. pylori* eradication and the first ER had not been detailed in several studies, and thus the prolonged infection status might potentially influence the preventive effectiveness. Finally, other confounders for gastric cancer risk such as infection-attributable oncoviruses, family history of gastric cancer, and ethnicity had not been considered in the present investigation (65–68).

## Conclusion

In summary, the present meta-analysis can further evidence that the *H. pylori* infection might potentially increase the post-ER incidence of MGC or dysplasia, but post-ER eradication can decrease the risk of MGC or dysplasia. Specifically, post-ER screening and eradication of *Helicobacter pylori* have preventive effectiveness on MGC, and the protocol should be recommended to all the post-ER patients. Moreover, larger-scale RCTs with longer follow-up duration are still warranted to define the best cost-effective interval and length for endoscopic surveillance among *H. pylori*-infected individuals after ER.

## Data availability statement

The original contributions presented in the study are included in the article/Supplementary material, further inquiries can be directed to the corresponding author.

## Author contributions

T-HY: Conceptualization, Data curation, Formal analysis, Investigation, Methodology, Software, Writing – original draft, Writing – review & editing. DB: Data curation, Investigation, Methodology, Writing – review & editing. KL: Data curation, Methodology, Writing – review & editing. W-HZ: Data curation, Investigation, Methodology, Supervision, Writing – review & editing. X-ZC: Conceptualization, Data curation, Funding acquisition, Investigation, Methodology, Project administration, Resources, Software, Supervision, Validation, Writing – original draft, Writing – review & editing. J-KH: Funding acquisition, Resources, Supervision, Writing – review & editing.
