# Supplementary material for: Helicobacterpylori eradication following endoscopic resection might prevent metachronous gastric cancer: a systematic review and meta-analysis of studies from Japan and Korea
Source: Front Med (Lausanne). 2024 Sep 2;11:1393498. doi: 10.3389/fmed.2024.1393498 (PMC11402824; doi:10.3389/fmed.2024.1393498)
Supplement: Supplementary file 1 [file Table_1.pdf]

Helicobacter pylori Eradication Following Endoscopic Resection Might Prevent Metachronous Gastric Cancer: A Systematic Review and Meta-analysis of Studies From Japan and Korea

Authors: Tian-Hang Yu,[1] Dan Bai,[2] Kai Liu,[1] Wei-Han Zhang,[1] Xin-Zu Chen,[1,3,4] Jian-Kun Hu;[1] on behalf of the SIGES Research Group

Affiliations:

1. Gastric Cancer Center & Gastric Cancer Laboratory, Department of General Surgery, West China Hospital, Sichuan University, Chengdu, China
2. Department of Day Surgery, General Practice Medical Center, West China Hospital, Sichuan University, Chengdu, China
3. Ya'an Cancer Prevention and Control Center, Ya'an People's Hospital - West China Ya'an Hospital, Sichuan University, Ya'an, China
4. Ya'an Key Laboratory for High Altitude Medicine, Ya'an People's Hospital - West China Ya'an Hospital, Sichuan University, Ya'an, China

Correspondence: Xin-Zu Chen, Gastric Cancer Center and Laboratory of Gastric Cancer, West China Hospital, Sichuan University, Guo Xue Xiang 37, Chengdu, Sichuan, China. Email: chenxinzu@scu.edu.cn

Supplementary Table 1. The brief characteristics and extracted data of the included studies.

Study	Year	Country	Design	Follow-up period (range) (Negative)/Eradicated/Persistent	Primary disease	MGL outcome	Event/total number		
							Negative	Eradicated	Persistent
Yoo, H. W.	2023	Korea	Cohort	5.9 years (range 3.9-7.8)/5.3 years (range 3.4-7.4)	Dysplasia	MGC<br>Dysplasia,<br>MGC and dysplasia	/<br>/<br>/	747/34,209<br>154/34,209<br>901/34,209	1,659/35,513<br>782/35,513<br>2,441/35,513
Noh, C. K.	2023	Korea	Cohort	25 months (IQR: 18-35) / 39 months (IQR: 27-57)	Dysplasia	MGC,<br>Dysplasia,<br>MGC and dysplasia	/<br>/<br>/	3/163<br>3/163<br>6/163	9/510<br>13/510<br>22/510
Nakata, R.	2021	Japan	Cohort	40.0 months (IQR 26.0-59.8）/38.5 months (IQR 25.0-66.5)	EGC	MGC	/	5/126	17/126
Kato, M.	2021	Japan	Cohort	3.5 years (range 1.1–14.8)	EGC	MGC	/	52/294	35/189
Ikeda, R.	2021	Japan	Cohort	39.8 months (range 18–180)	EGC	MGC	19/61	34/315	100/562
Okada, K.	2019	Japan	Cohort	4.1 years (range 3.0-5.7)/ 4.1years (range 2.9-5.6)	EGC	MGC	/	27/174	33/174
Han, S. J.	2018	Korea	Cohort	60, 61, and 60 months (range 12-122)	EGC	MGC	20/157	12/212	18/196
Choi, J. M.	2018	Korea	RCT	71.7 months(range 44.2-91.7)/70.6 months (range 39.3-87.4)	EGC and dysplasia	MGC	/	18/437	36/440
Kwon, Y.	2017	Korea	Cohort	47.7 months (range18.4–125)	EGC	MGC and dysplasia	11/56	33/368	8/27
Chung, C. S.	2017	Korea	Cohort	24 months (range:6 to 75)	EGC	MGC and dysplasia	/	17/167	7/18
Kim, S. B.	2016	Korea	Cohort	36.18 months (±26.74)/32.78 months (±23.72)/33.29 months (±25.93)	EGC	MGC	7/95	3/120	1/42
Shin, S. H.	2015	Korea	Cohort	53.0 months (range 26.3–85.7) /58.3 months (range 24.3–85.9) /57.2 months (range 28.1–85.2)	Dysplasia	MGC,<br>Dysplasia	4/124<br>15/124	4/122<br>6/122	3/36<br>4/36
Jung, S.	2015	Korea	Cohort	42.6 months	EGC and dysplasia	MGC and dysplasia	4/366	10/169	21/506
Kwon, Y. H.	2014	Korea	Cohort	40 months (36–60)/ 44 months (37–60)	EGC	MGC,<br>Dysplasia	/<br>/	10/214<br>8/214	10/69<br>3/69

Abbreviations: EGC, early gastric cancer; IQR, inter-quartile range; MGC, metachronous gastric cancer; RCT, randomized controlled trial.

Supplementary Table 1. The brief characteristics and extracted data of the included studies. (continued)

Study	Year	Country	Design	Follow-up period (range) (Negative)/Eradicated/Persistent	Primary disease	MGL outcome	Event/total number		
							Negative	Eradicated	Persistent
Kim, Y. I.	2014	Korea	Cohort	4.1 years (range 3.1–5.8)/5.3years (range 4.0–8.3)/4.6 years (range 3. 1–6.1)	EGC	MGC	13/218	2/49	16/107
Choi, J.	2014	Korea	Open label RCT	36.7 months (range 23.9–54.8) /36.2 months (range 23.8–54.0)	EGC and dysplasia	MGC	/	7/213	11/212
						Low-grade dysplasia	/	3/176	6/174
						High-grade dysplasia	/	0/50	0/55
						MGC and dysplasia	/	10/439	17/441
Bae, S. E.	2014	Korea	Cohort	59.0 months (range 24 – 116)/ 59 months (range 24 – 137) /61.5 months(range 24 – 114)	EGC	MGC	17/340	34/485	24/182
Maehata, Y.	2012	Japan	Cohort	3.0years (range 1.1-11.1)/ 3.0 years (range 1.1-10.3)	EGC	MGC	/	15/177	13/91
Shiotani, A.	2008	Japan	Cohort	33 months (range 24–48)	EGC	MGC	1/9	9/80	1/11
Fukase, K.	2008	Japan	Open label RCT	3 years	EGC	MGC	/	9/255	24/250
Nakagawa, S.	2006	Japan	Cohort	2.6years (range 0.5-8)/1.8years (range 0.5-12)	EGC	MGC	/	8/356	129/2,479

Abbreviations: EGC, early gastric cancer; IQR, inter-quartile range; MGC, metachronous gastric cancer; RCT, randomized controlled trial.

Supplementary Table 2. Newcastle-Ottawa scores for included cohort studies. #

		Selection				Comparability	Outcome			
Study	Year	Representativeness of the exposed cohort	Selection of the non-exposed cohort	Ascertainment of exposure	Demonstration that outcome of interest was not present at start of study	Comparability of cohorts on the basis of the design or analysis	Assessment of outcome	Was follow-up long enough for outcomes to occur?	Adequacy of follow-up cohorts	Score
Yoo, H. W.	2023	1	1	1	1	1	1	1	1	8
Noh, C. K.	2023	1	1	1	1	1	1	1	0	7
Nakata, R.	2021	1	1	1	1	1	1	1	0	7
Kato, M.	2021	1	1	0	1	1	1	1	0	6
Ikeda, R.	2021	1	0	1	1	1	1	1	0	6
Okada, K.	2019	1	1	1	1	1	1	1	1	8
Han, S. J.	2018	1	1	0	1	1	1	1	0	6
Kwon, Y.	2017	1	1	1	1	1	1	1	0	7
Chung, C. S.	2017	1	1	1	1	1	1	1	0	7
Mori, G.	2016	1	1	1	1	1	1	1	1	8
Kim, S. B.	2016	1	1	1	1	0	1	1	0	6
Shin, S. H.	2015	1	1	1	1	0	1	1	0	6
Jung, S.	2015	1	1	1	1	0	1	1	1	7
Kwon, Y. H.	2014	1	1	1	1	1	1	1	1	8
Kim, Y. I.	2014	1	1	1	1	1	1	1	1	8
Bae, S. E.	2014	1	1	1	1	1	1	1	1	8
Maehata, Y.	2012	1	1	1	1	1	1	1	1	8
Shiotani, A.	2008	1	1	1	1	0	1	1	1	7
Nakagawa, S.	2006	1	1	1	1	1	1	1	1	8

Studies were awarded a maximum of two points for the category “Comparability of cohorts on the basis of the design or analysis”; but for other categories, only a maximum of one point can be given.

Supplementary Table 3. Cochrane risk of bias tool for included RCTs. #

Study	Year	Random sequence generation (selection bias)	Allocation concealment (selection bias)	Blinding of participants and personnel (performance bias)	Blinding of outcome assessment (detection bias)	Incomplete outcome data (attrition bias)	Selective reporting (reporting bias)	Other bias
Choi, J. M.	2018	1	1	1	?	0	1	1
Choi, J.	2014	1	?	0	?	0	?	1
Fukase, K.	2008	1	?	0	1	0	?	1

Reviewers' judgment about each risk of bias for each included RCT, "0" for low risk of bias, "1" for high risk of bias, "?" for unclear risk of bias.

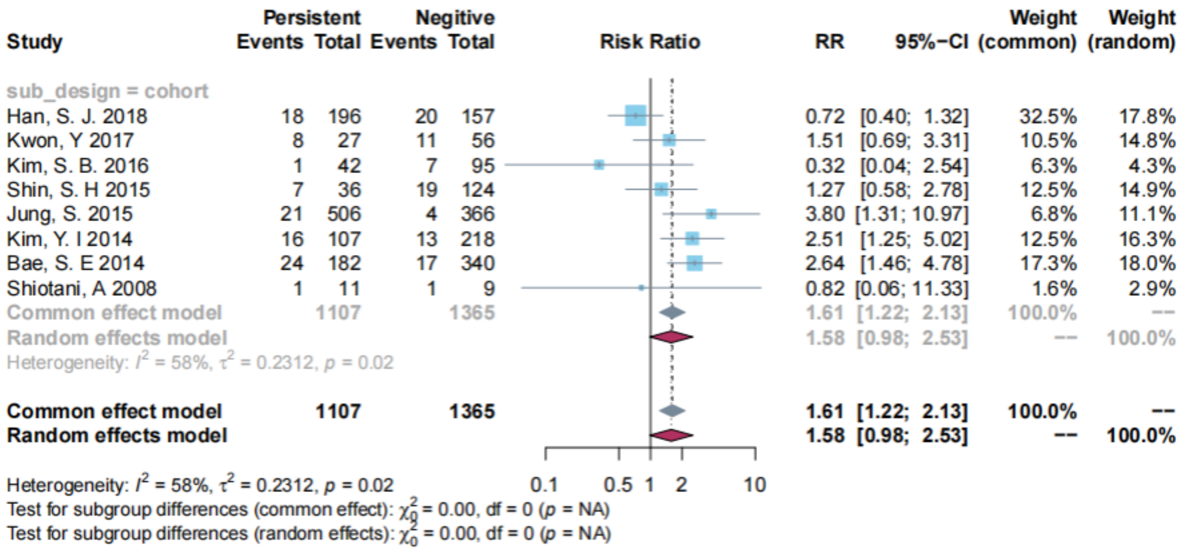

Supplementary Figure 1. Forest plot of subgroup analysis between persistent and negative patients stratified by study design.

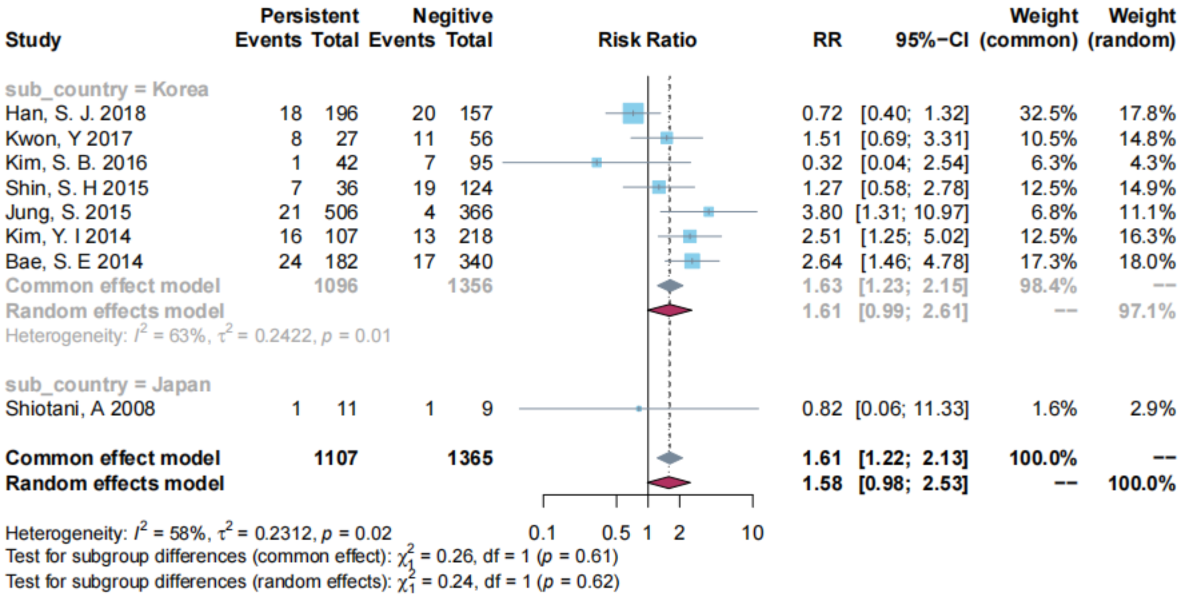

Supplementary Figure 2. Forest plot of subgroup analysis between persistent and negative patients stratified by country.

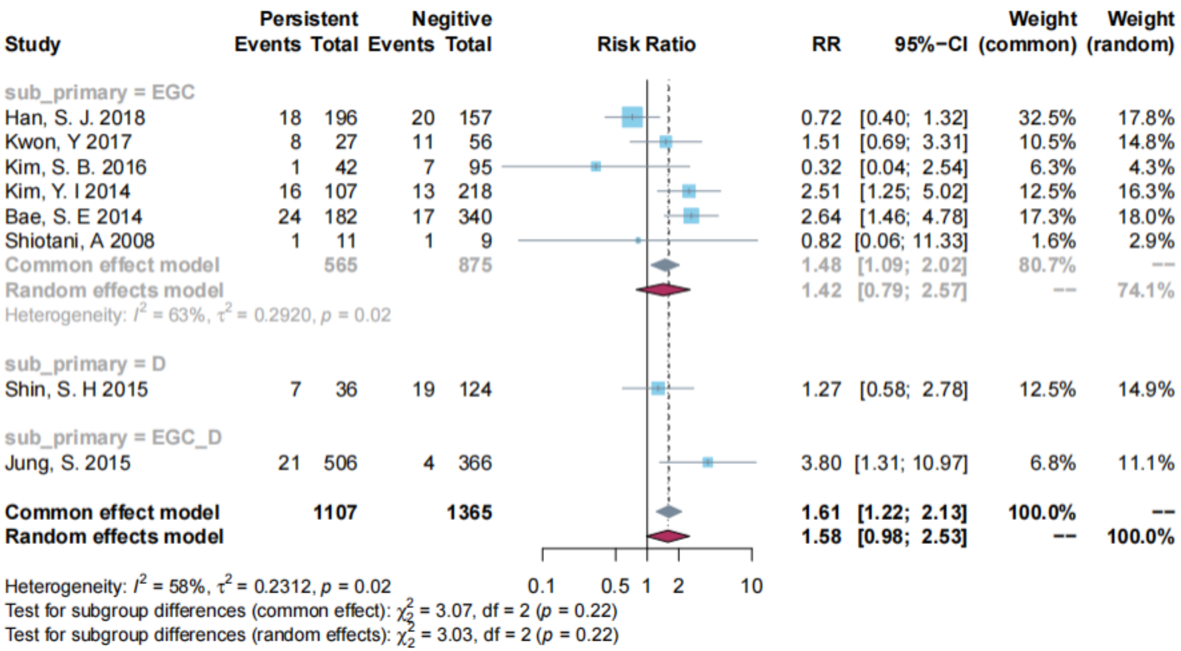

Supplementary Figure 3. Forest plot of subgroup analysis between persistent and negative patients stratified by primary disease.

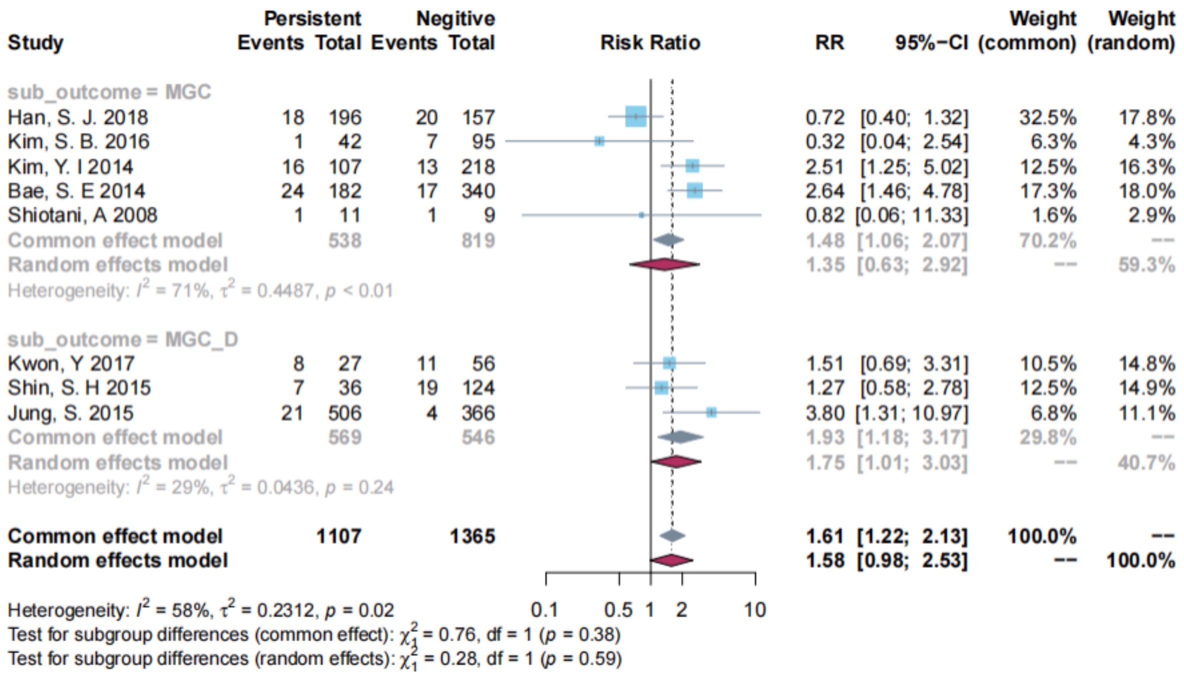

Supplementary Figure 4. Forest plot of subgroup analysis between persistent and negative patients stratified by outcome pattern.

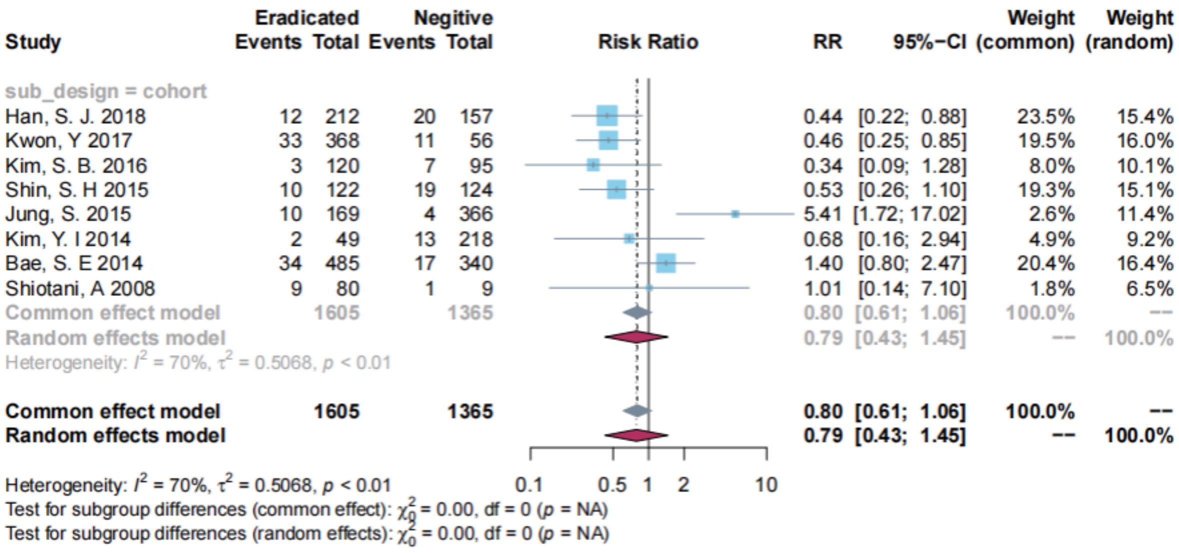

Supplementary Figure 5. Forest plot of subgroup analysis between eradicated and negative patients stratified by study design.

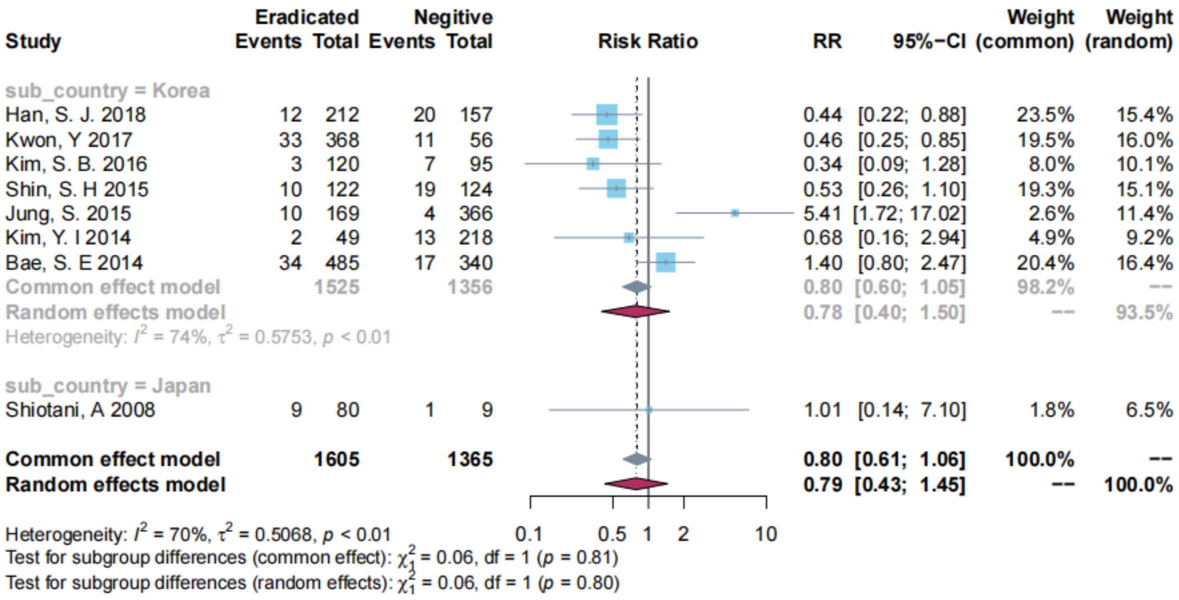

Supplementary Figure 6. Forest plot of subgroup analysis between eradicated and negative patients stratified by country.

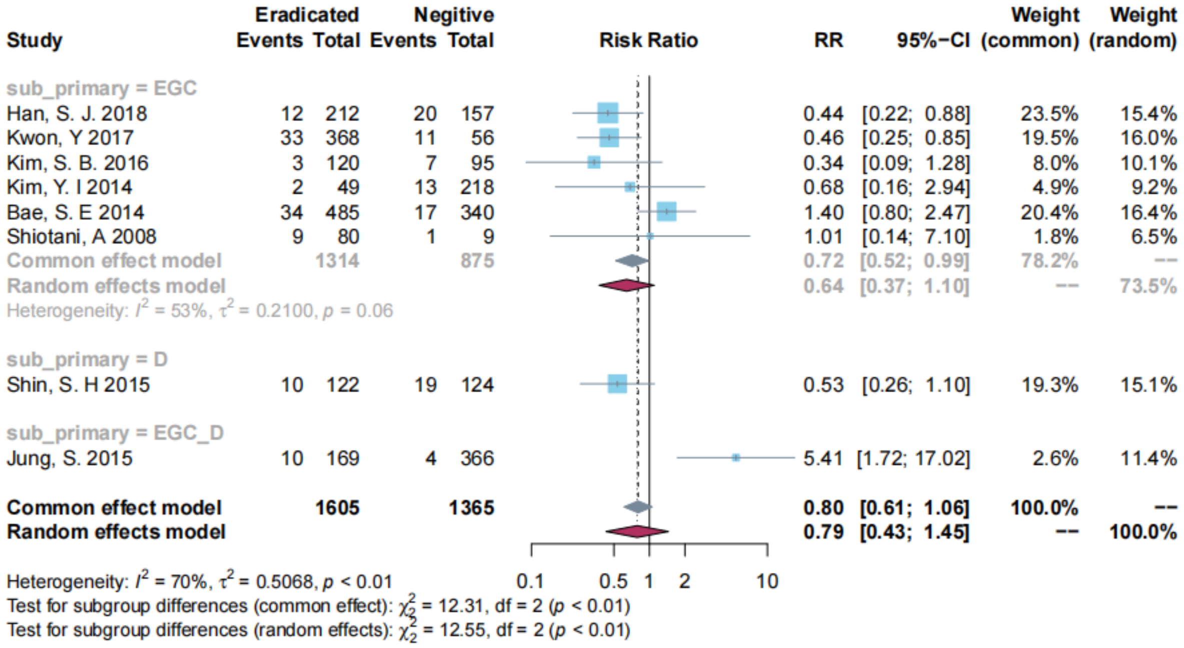

Supplementary Figure 7. Forest plot of subgroup analysis between eradicated and negative patients stratified by primary disease.

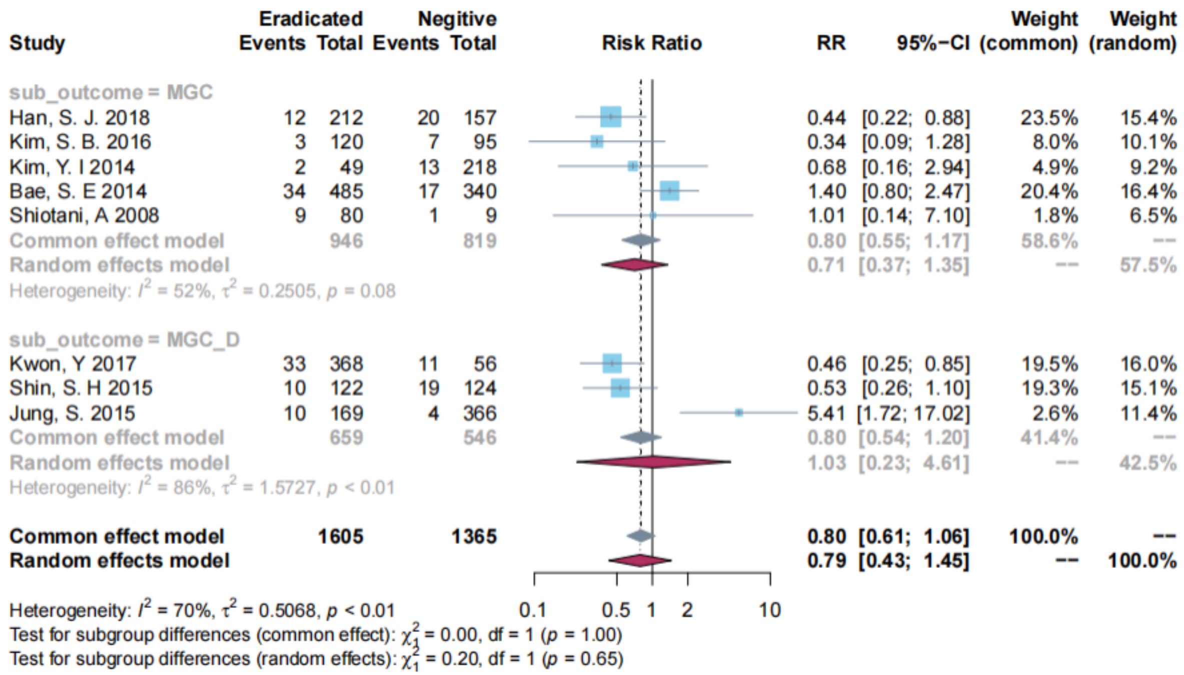

Supplementary Figure 8. Forest plot of subgroup analysis between eradicated and negative patients stratified by outcome pattern.

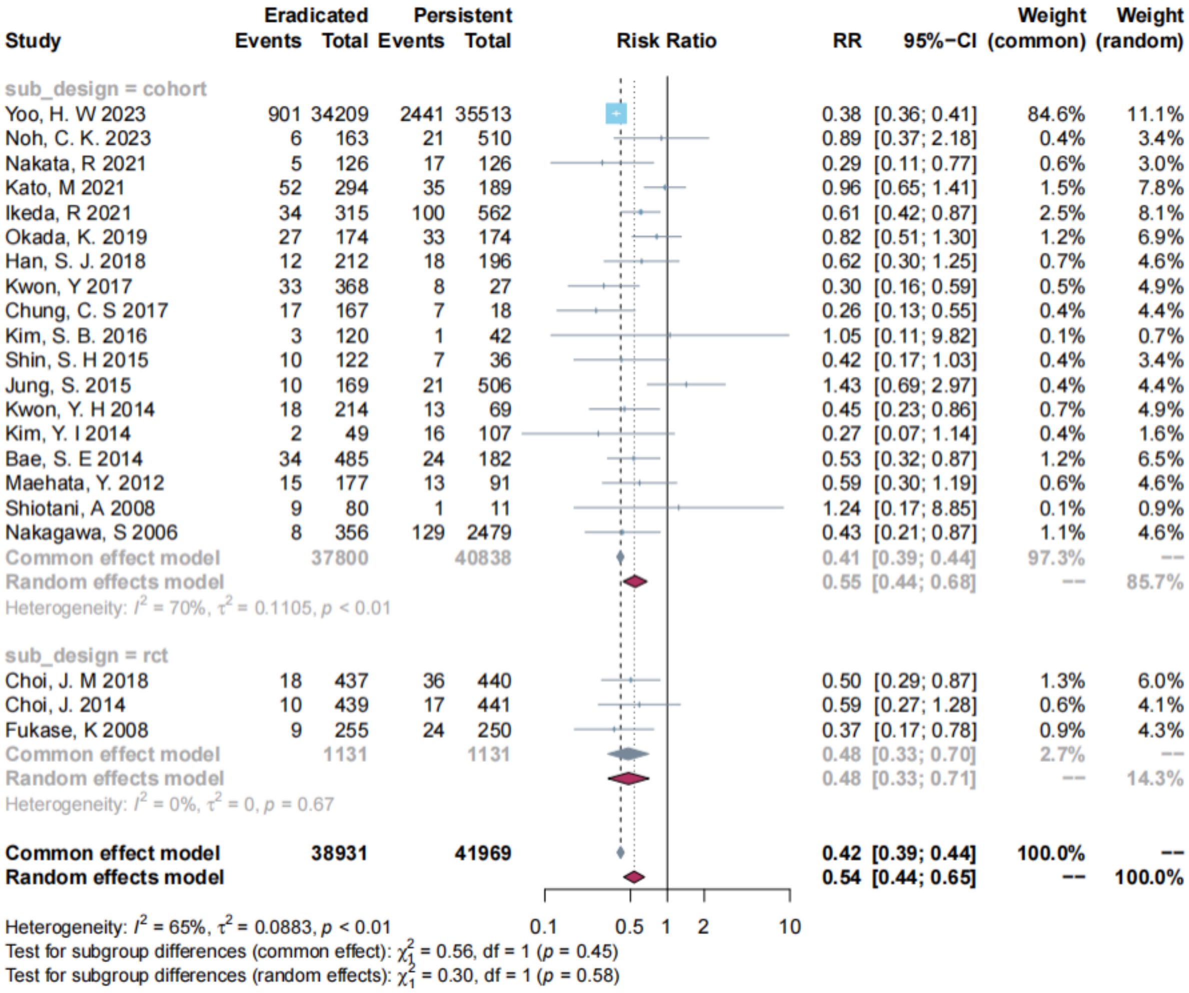

Supplementary Figure 9. Forest plot of subgroup analysis between eradicated and persistent patients stratified by study design.

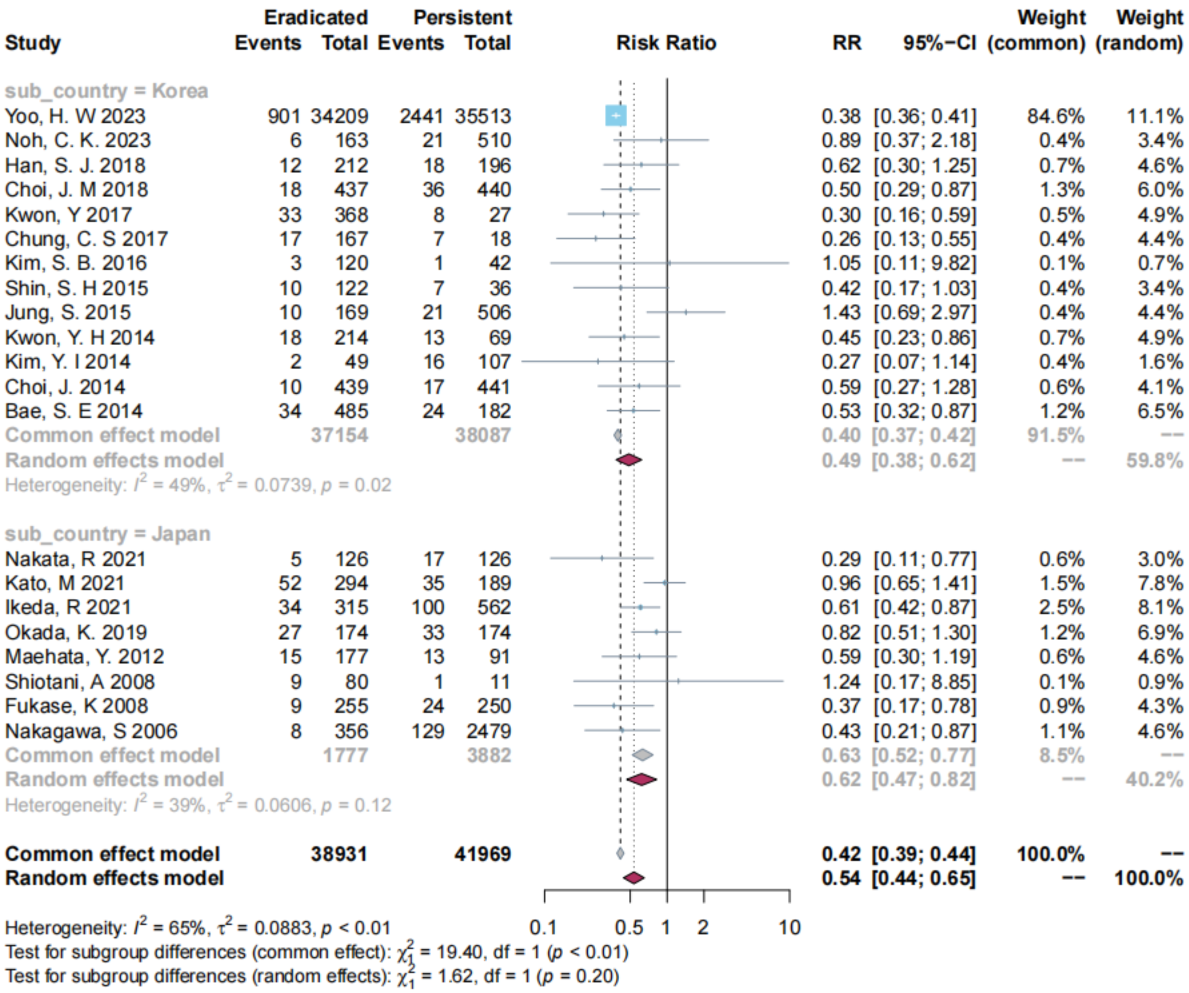

Supplementary Figure 10. Forest plot of subgroup analysis between eradicated and persistent patients stratified by country.

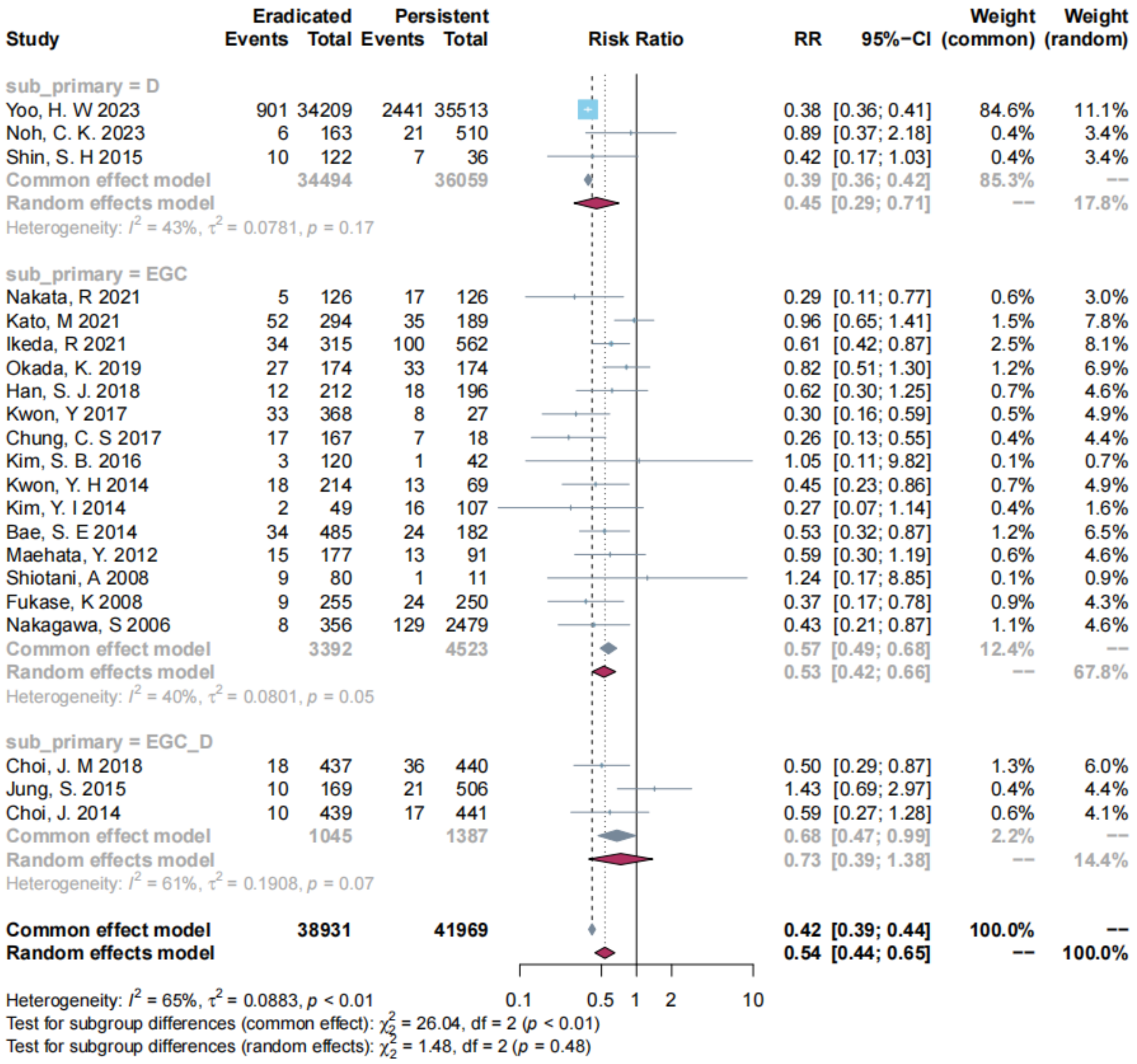

Supplementary Figure 11. Forest plot of subgroup analysis between eradicated and persistent patients stratified by primary disease.

Study	Eradicated Events	Eradicated Total	Persistent Events	Persistent Total	RR	95%−CI	Weight (common)	Weight (random)
sub_outcome = MGC_D								
Yoo, H. W 2023	901	34209	2441	35513	0.38	[0.36; 0.41]	84.6%	11.1%
Noh, C. K. 2023	6	163	21	510	0.89	[0.37; 2.18]	0.4%	3.4%
Kwon, Y 2017	33	368	8	27	0.30	[0.16; 0.59]	0.5%	4.9%
Chung, C. S 2017	17	167	7	18	0.26	[0.13; 0.55]	0.4%	4.4%
Shin, S. H 2015	10	122	7	36	0.42	[0.17; 1.03]	0.4%	3.4%
Jung, S. 2015	10	169	21	506	1.43	[0.69; 2.97]	0.4%	4.4%
Kwon, Y. H 2014	18	214	13	69	0.45	[0.23; 0.86]	0.7%	4.9%
Choi, J. 2014	10	439	17	441	0.59	[0.27; 1.28]	0.6%	4.1%
Common effect model		35851		37120	0.39	[0.36; 0.42]	88.0%	--
Random effects model					0.49	[0.33; 0.70]	--	40.4%
Heterogeneity: $I^2 = 62\%$, $\tau^2 = 0.1694$, $p < 0.01$								
sub_outcome = MGC								
Nakata, R 2021	5	126	17	126	0.29	[0.11; 0.77]	0.6%	3.0%
Kato, M 2021	52	294	35	189	0.96	[0.65; 1.41]	1.5%	7.8%
Ikeda, R 2021	34	315	100	562	0.61	[0.42; 0.87]	2.5%	8.1%
Okada, K. 2019	27	174	33	174	0.82	[0.51; 1.30]	1.2%	6.9%
Han, S. J. 2018	12	212	18	196	0.62	[0.30; 1.25]	0.7%	4.6%
Choi, J. M 2018	18	437	36	440	0.50	[0.29; 0.87]	1.3%	6.0%
Kim, S. B. 2016	3	120	1	42	1.05	[0.11; 9.82]	0.1%	0.7%
Kim, Y. I 2014	2	49	16	107	0.27	[0.07; 1.14]	0.4%	1.6%
Bae, S. E 2014	34	485	24	182	0.53	[0.32; 0.87]	1.2%	6.5%
Maehata, Y. 2012	15	177	13	91	0.59	[0.30; 1.19]	0.6%	4.6%
Shiotani, A 2008	9	80	1	11	1.24	[0.17; 8.85]	0.1%	0.9%
Fukase, K 2008	9	255	24	250	0.37	[0.17; 0.78]	0.9%	4.3%
Nakagawa, S 2006	8	356	129	2479	0.43	[0.21; 0.87]	1.1%	4.6%
Common effect model		3080		4849	0.60	[0.51; 0.71]	12.0%	--
Random effects model					0.60	[0.49; 0.73]	--	59.6%
Heterogeneity: $I^2 = 16\%$, $\tau^2 = 0.0313$, $p = 0.29$								
**Common effect model**		**38931**		**41969**	**0.42**	**[0.39; 0.44]**	**100.0%**	**--**
**Random effects model**					**0.54**	**[0.44; 0.65]**	**--**	**100.0%**

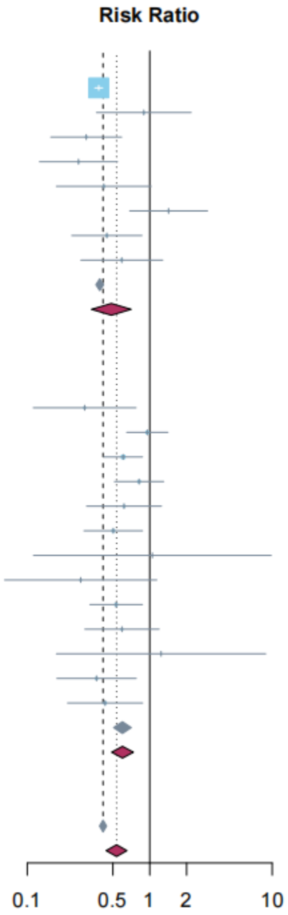

Heterogeneity: $I^2 = 65\%$, $\tau^2 = 0.0883$, $p < 0.01$
Test for subgroup differences (common effect): $\chi^2_1 = 21.21$, df = 1 ($p < 0.01$)
Test for subgroup differences (random effects): $\chi^2_1 = 0.94$, df = 1 ($p = 0.33$)

Supplementary Figure 12. Forest plot of subgroup analysis between eradicated and persistent patients stratified by outcome pattern.

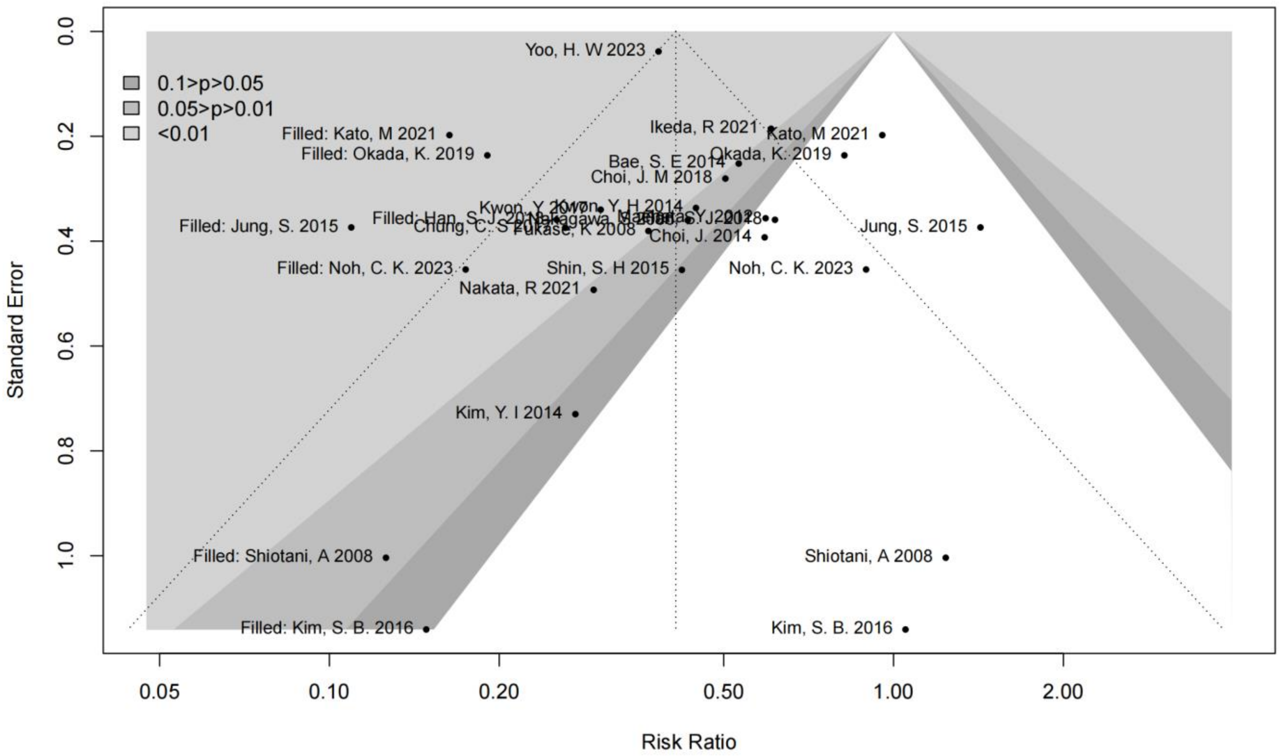

Supplementary Figure 13. Contour-enhanced meta-analysis funnel plots after Duval and Tweedie trimand fill method between eradicated and persistent patients
